# Choice overload constrains mate choice independent of energetic masking

**DOI:** 10.1016/j.isci.2026.117086

**Published:** 2026-08-12

**Authors:** Claire T. Hemingway, André Vieira Rodrigues, Alejandro Vélez, Jessie C. Tanner

**Affiliations:** 1 Department of Ecology & Evolutionary Biology, University of Tennessee, Knoxville, TN 37916, USA; 2 Department of Psychology & Neuroscience, University of Tennessee, Knoxville, TN 37916, USA; 3 Collaborative for Animal Behavior, University of Tennessee, Knoxville, TN 37916, USA; 4 Center for Animal Behavior, Smithsonian Tropical Research Institute, Balboa, Ancón, Panama

**Keywords:** choice overload, energetic masking, background noise, animal communication, sexual selection

## Abstract

Mate choice requires receivers to detect, remember, and compare signals across multiple potential mates. Strong preferences in simple contexts often weaken when receivers encounter numerous signalers, potentially due to energetic masking, in which overlapping signals impair discrimination, or choice overload, in which evaluating too many options exceeds cognitive capacity. Although energetic masking is well-documented, we know little about choice overload in nonhumans. We investigated both mechanisms in Cope’s gray treefrogs (*Hyla chrysoscelis*) using playback experiments that independently manipulated the number of non-overlapping calls and background chorus noise. Females chose between a longer “target” call and one, three, or seven shorter “distractor” calls with and without background noise. As the number of calls increased, females became less accurate, took longer to decide, and more frequently deferred choosing. These results demonstrate that cognitive limits on information processing can subvert mate choice, even without sensory interference, highlighting an underappreciated constraint on sexual selection.

## Highlights

• Accuracy for preferred male calls in treefrogs declined as choice set sizes increased
• Females also increased decision time and choice deferral with more calling males
• Noise altered these decision dynamics and buffered choice overload effects
• We show that cognitive limits shape mate choice beyond acoustic masking effects

## Graphical abstract

**Figure:**
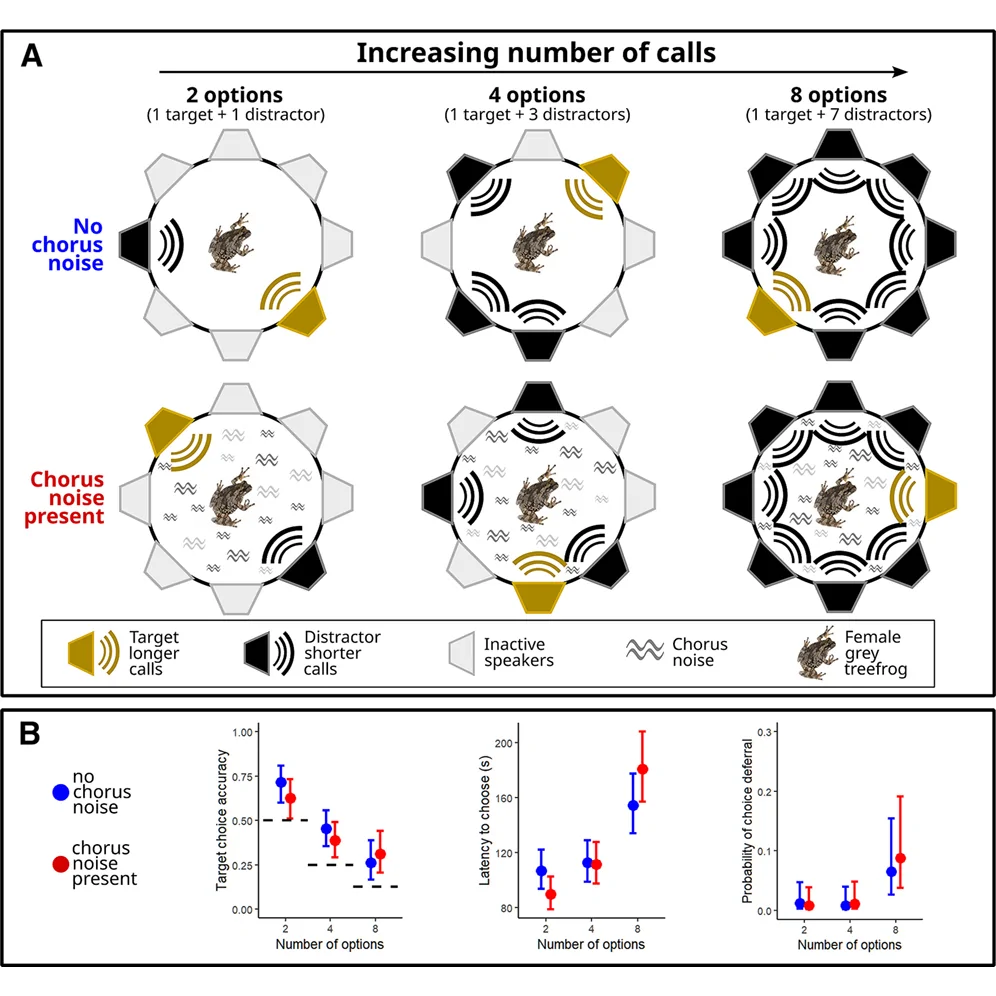

## Teaser

Biological sciences; Evolutionary ecology; Animal behavior

## Introduction

For many animals, mate choice poses a high-stakes information-processing problem: receivers must detect and recognize mating signals, retain this information while sampling multiple signalers, and compare alternatives to reach a decision.^1^^,^^2^ This is a relatively straightforward task in simple scenarios where receivers evaluate only one or two signalers, which is typical in most studies of mate choice in animals.^3^ In natural settings, however, many animals signal for mates in groups,^4^ producing conspicuous signals that overlap in time and space, and females must navigate these conditions while operating under both sensory and cognitive constraints.^5^ In studies where females have access to many options, preferences that are clear in simpler scenarios often deteriorate.^6^^,^^7^^,^^8^ Because mate choice directly shapes reproductive success, such deterioration has far-reaching implications for how we understand and model sexual selection pressures. Understanding how receivers navigate complex signaling environments is, therefore, critical to explaining variation in mate preferences and the evolutionary dynamics of sexual signaling. However, mate-choice studies often overlook sensory and cognitive constraints that limit the ability to detect, discriminate among, and evaluate all available options.

Two broad mechanisms may explain how signal competition shapes receiver perception, leading to weaker preferences as more signalers aggregate and display to females. The first is energetic masking, in which signals overlap in time or frequency, saturating sensory filters and making it difficult for receivers to distinguish relevant features of a target signal.^9^ Energetic masking is a well-documented and long-recognized constraint in acoustically communicating animals,^10^ including humans^9^ and other mammals,^11^ birds,^12^ frogs,^13^ and insects.^14^ The impact of masking is particularly evident when multiple signalers broadcast within the same frequency ranges.^9^^,^^15^ A second, rarely considered mechanism is choice overload, in which the complexity of the choice problem exceeds an individual’s cognitive resources when too many options are simultaneously available, reducing several aspects of receiver performance.^16^ At a cognitive level, the demands of evaluating multiple alternatives can exceed an animal’s processing limits, leading individuals to make suboptimal decisions, take longer to reach a decision, or even forgo a decision altogether. Choice overload is well-documented in humans, especially in economic decision-making^17^; however, growing evidence suggests that other animals face similar cognitive constraints when confronted with too many options (bees,^18^ ants,^19^ mosquitos,^20^ field crickets,^8^ and frogs^7^). While masking occurs at the peripheral sensory level, preventing signals from being accurately detected, choice overload arises from limits on cognitive processing, where information is received but not effectively integrated. This overload could disrupt processing at multiple stages, including perceptual encoding, stimulus evaluation, or comparison across alternatives.

Although energetic masking and choice overload disrupt sensory processing in distinct ways, they often co-occur in natural environments, making it challenging to tease apart their individual effects on decision-making. In frogs and other acoustically communicating animals, both may play important roles in female decision-making. During a breeding season, males gather in dense choruses, sometimes numbering in the hundreds and often including multiple species, where males call to attract females and deter rivals. These advertising calls can be remarkably loud, reaching a peak sound pressure level (SPL) of up to 108 dB at 50 cm.^21^ When many individuals form breeding aggregations, the sum of individual signaling interactions generates substantial background noise.^8^^,^^22^^,^^23^ This high level of background noise and overlap may lead to auditory masking, impairing signal detection, discrimination, recognition, or localization.^15^^,^^24^ A handful of mate-choice studies in frogs have shown that female selectivity for preferred acoustic features of individual calls generally weakens as the number of options increases beyond two.^7^^,^^16^^,^^25^^,^^26^^,^^27^^,^^28^ Such findings may be explained by the noisy conditions produced by chorusing males, leading to auditory masking.^15^^,^^24^ At the same time, the sheer abundance of calling males may cause choice overload, as females are confronted with more options than they can efficiently evaluate.

While most research has focused on energetic masking, cognitive constraints also play an important role in shaping choice behavior across taxa.^16^^,^^29^ However, few studies have explicitly separated these mechanisms. We used Cope’s gray treefrogs (*Hyla chrysoscelis* = *Dryophytes chrysoscelis*) to test whether female preference expression depends on increasing competing stimuli, and if so, whether this results from the number of males available (choice overload), the background chorus produced by multiple calling males (masking), or both. This system is well-suited for disentangling these mechanisms because individual frog calls and choruses can be simulated to experimentally manipulate the number of calling males independent of background noise. By preventing signal overlap, we induced the conditions hypothesized to cause choice overload while eliminating acoustic masking. Conversely, we introduced background noise in conditions with few calls, where masking is unlikely to naturally occur. Although females in this system can detect multiple males within a limited spatial range and must sample calls across time, the precise sampling rule (e.g., sequential comparison vs. threshold-based decision-making) remains unclear. Here, we focus on how the number of available options and background noise influence decision-making regardless of the specific sampling strategy.

We gave female frogs choices based on call duration, a trait for which receivers typically show strong directional preferences for longer calls.^30^^,^^31^ In each test, we presented receivers with a choice between one long “target” call and one, three, or seven shorter “distractor” calls. We quantified declines in decision performance as decreased choice accuracy (preferential selection of the target), increased decision latency (time elapsed before approaching a call), and increased choice deferral (the probability of *not* making a decision during the allotted time). We evaluated the roles of background noise and choice overload using a within-subjects design: females were tested in each choice-set size with and without background noise. If noise plays a more important role in shaping receiver preferences, decision performance should decline in the noise condition relative to the quiet condition, regardless of choice-set size. If choice overload is more important, decision performance should decline as set size increases, independent of noise. Finally, both energetic masking and choice overload may contribute additively or interactively, with performance lowest under noise and declining either proportionately or disproportionately as the number of options increases.

## Results

### Accuracy

**Figure 1:**
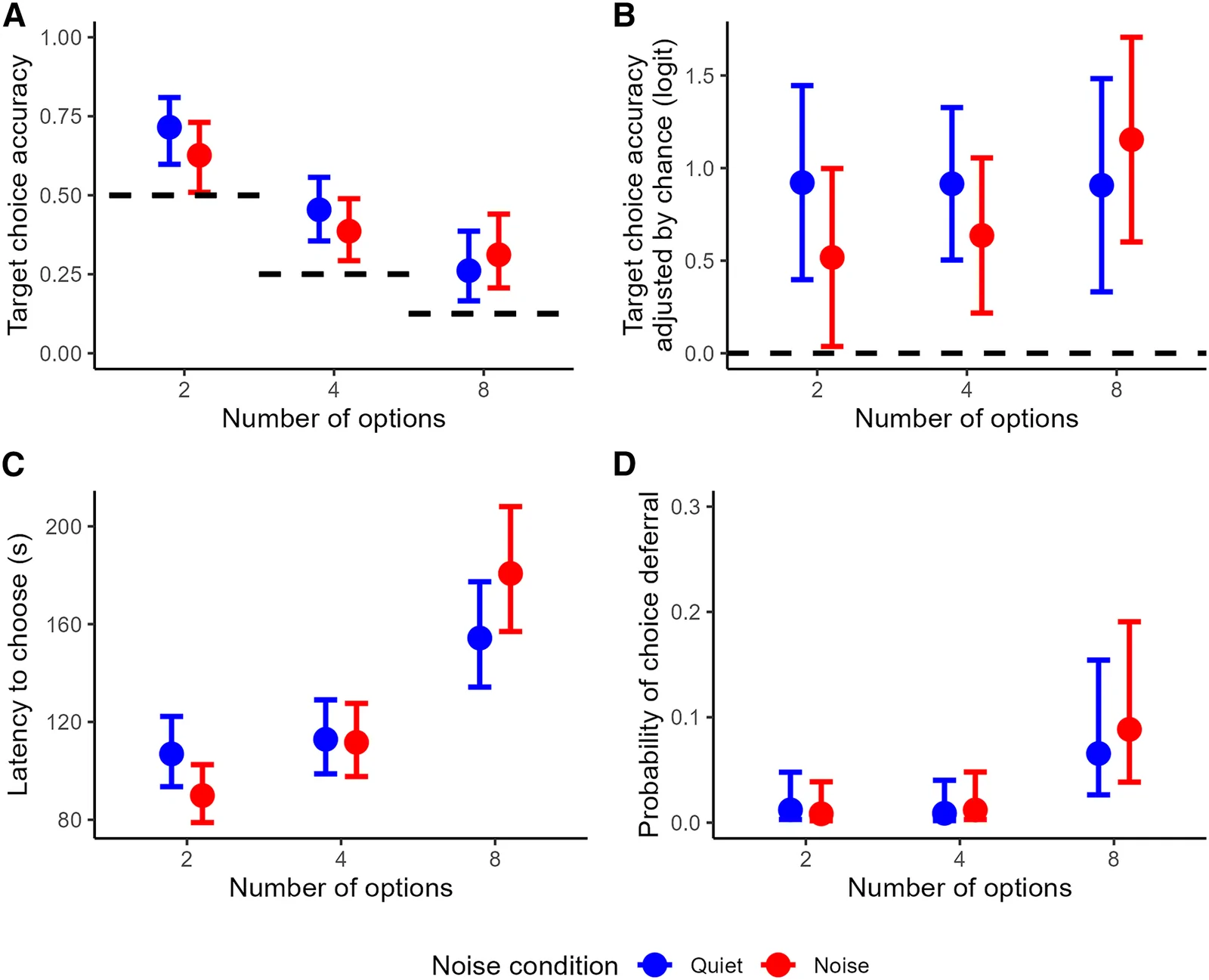
Female choice behaviors under varying numbers of options and noise conditions (A) Choice accuracy, measured as the probability of choosing the target stimulus; dashed lines indicate the chance expectation for each number of options. (B) Choice accuracy adjusted relative to chance expectation using Monte Carlo simulations; shown on the logit scale. The dashed line marks the chance level. (C) Latency, in seconds, to make a choice. (D) Probability of choice deferral or not making a choice in the 10 min trial period. Graphics show means with 95% confidence intervals. Treatments are color-coded (blue: quiet; red: noise) (*N* = 60 females).

Choice accuracy declined as the number of options increased (Figure 1A; Table S1), but receivers continued to choose longer calls more often than expected by chance. In the quiet condition, females chose the target speaker in 83 of 108 (76.9%) two-choice trials, 51 of 108 (47.2%) four-choice trials, and 20 of 97 (20.6%) eight-choice trials. In the noise condition, they chose the target speaker in 77 of 112 (68.8%) two-choice trials, 45 of 108 (41.7%) four-choice trials, and 23 of 94 (24.5%) eight-choice trials. The decline in accuracy was steep in the quiet condition, with significant drops from two to four options and from four to eight options (contrast_(2 vs. 4)_quiet_ = 3.02, SE = 0.984, *p* = 0.001; contrast_(4 vs. 8)_quiet_ = 2.35, SE = 0.838, *p* = 0.016). In noise, accuracy leveled off beyond four options, showing no significant difference between four and eight options (contrast_(4 vs. 8)_noise_ = 1.39, SE = 0.485, *p* = 0.345).

Even though overall choice accuracy declined as the number of options increased, female performance relative to chance did not differ across option numbers in the quiet condition, after adjusting for differences in chance expectations (Figure 1B; Table S2). This suggests that, in quiet conditions, females do not choose randomly, regardless of choice set size. In contrast, noise reversed this pattern: adjusted accuracy above chance increased with option number and was highest in the eight-choice test relative to both the two-choice (MC P_(8 > 2)_noise_ = 0.954) and four-choice tests (MC P_(8 > 4)_noise_ = 0.929). Noise reduced choice accuracy relative to the quiet condition in the two-choice tests (MC P_(2_noise < 2_quiet)_ = 0.865) but tended to increase accuracy in the eight-choice tests (MC P_(8_noise > 8_quiet)_ = 0.728).

### Latency to choose

Receivers took longer to make decisions as the number of options increased, consistent with choice overload (Figure 1C; Table S3). Mean latency increased from 114.4 s in two-choice trials to 128.2 s in four-choice trials and 162.6 s in eight-choice trials in the quiet condition, and from 96.0 s to 121.0 s–183.3 s in the noise condition. Notably, noise reshaped this relationship: in two-choice trials, females actually made decisions faster in the noise condition than in the quiet condition, but as the number of options increased, noise appeared to exacerbate the effects of choice overload such that eight-choice trials took longer in noise than in quiet (contrast_(2 vs. 8):(quiet vs. noise)_ = 1.39, SE = 0.158, *p* = 0.010).

### Choice deferral

Females were increasingly likely to defer as the number of options increased, regardless of noise condition (Figure 1D; Table S4). In two-choice trials, females deferred in 7 of 220 trials; in four-choice trials, 7 of 216; and in eight-choice trials, 34 of 191. They deferred significantly more often in eight-choice trials compared to both two- and four-choice trials. This held true in both quiet (contrast_(2 vs. 8)_quiet_ = 0.174, SE = 0.111, *p* = 0.009; contrast_(4 vs. 8)_quiet_ = 0.128, SE = 0.090, *p* = 0.009) and noise conditions (contrast_(2 vs. 8)_noise_ = 0.087, SE = 0.061, *p* = 0.002; contrast_(4 vs. 8)_noise_ = 0.125, SE = 0.079, *p* = 0.002). In contrast, females were no more likely to defer in four-choice trials than in two-choice trials (contrast_(2 vs. 4)_quiet_ = 1.366, SE = 1.110, *p* = 0.702; contrast_(2 vs. 4)_noise_ = 0.697, SE = 0.567, *p* = 0.657). Background noise had no significant effect on the probability of deferral (Figure 1D).

### Temporal order and spatial position

While we randomized trial order between subjects, our *a priori* tests showed that trial order influenced female behavioral responses. Females became less selective and more responsive over time as they approached dropping their eggs. We therefore included trial number as a covariate in all models (Table S5). Temporal position of the target call affected choice accuracy in the two- and four-choice treatments, but not in the eight-choice tests, and was included only in the accuracy model (Table S6). In contrast, neither the spatial arrangement of active speakers (i.e., whether the target was spatially isolated or surrounded by distractors), nor angular separation between target and distractor speakers affected any response variable (Tables S7 and S8), and these factors were excluded from the final analyses.

## Discussion

We examined how choice set size and background noise influenced female treefrog mate choice decisions both separately and jointly by comparing conditions with an increasing number of non-overlapping calls in the presence and absence of background noise. We found that as the number of calling males increased, receivers became less likely to choose the preferred target signal, took longer to decide, and became more likely to defer their decision. Together, these findings provide support that choice overload exerted a strong influence on female decision-making. Background noise also appeared to have a subtle effect on performance, such that females decided faster and were slightly less accurate in noisy conditions when facing fewer options. However, they took longer to decide and were slightly more accurate in larger choice sets. These findings suggest that noise can impair decision-making, while also buffering the effects of choice overload in multiple-option scenarios. Importantly, these results differentiate between choice overload and energetic masking, two mechanisms that may affect the expression of female mating preferences.^16^^,^^32^ Our results highlight the importance of cognitive, rather than just perceptual, limitations in shaping receiver preferences. These cognitive limitations are particularly relevant when animals encounter many options and must choose in the presence of noise, a common occurrence among acoustically advertising animals.^6^^,^^14^^,^^15^^,^^33^

The choice overload demonstrated here is particularly striking given the relatively large difference in call duration between the target and distractor calls. We intentionally designed this difference to be biologically meaningful and to avoid call overlap in time in all treatments. The duration of our target call is close to the population mean, while the duration of the distractor call is approximately one standard deviation below the mean. Previous studies with *H. chrysoscelis* and its sister taxon *Hyla versicolor* show that such differences between alternative calls elicit strong preferences when females are choosing between only two calling males in quiet conditions.^30^^,^^31^^,^^32^^,^^34^^,^^35^ Such strong preferences, however, attenuate in environments with more than two signalers.^36^^,^^37^ It has been suggested that the weakening of this preference is not due to the noise generated by more signaling males, but rather to the disruption of the temporal structure of calls when those of various males overlap.^36^ Accordingly, our results show that adding noise does not attenuate preference when females choose between two males. However, our results also show that, as the number of non-overlapping distractor calls increases, females become less likely to exert their preference for the target call. Our experimental design precluded disruption of the temporal structure of the calls in the quiet condition and provided support for the hypothesis that choice overload reduces the strength of female preference even when signal differences are pronounced and signals are not overlapping. Furthermore, because receivers exert selection on multiple call components that can carry information of differing value to receivers,^31^^,^^38^ choice overload could broadly influence the evolution of multiple signaling traits. Future studies could explore whether attributes that encode information about individuals^39^ or species recognition^40^^,^^41^ are similarly affected, or whether such traits are more resilient to choice overload.

Further support for the role of choice overload in these decision contexts comes from two other recent studies of field crickets and treefrogs, in which preferences for male calls diminish in larger choruses.^7^^,^^8^ In natural choruses, male signals often overlap, introducing both energetic masking and increased cognitive demands. Because the factors often co-occur, however, the two mechanisms could not previously be separated. In our study, we isolated the role of choice overload by presenting calls sequentially and found that performance declines even in the absence of overlapping signals, suggesting that choice overload alone can impair decision-making. Taken together with previous findings, this suggests that choice overload may weaken selection on call properties in natural settings, although in real choruses it likely operates alongside masking effects when signals overlap. If so, male reproductive success may be shaped not only by the acoustic properties of individual calls but also by the females’ cognitive demands associated with the complexity of the choice environment. Future work should explore the relative contribution of choice overload and energetic masking on female preferences in lekking species with temporally structured mating signals.

In addition to reduced preference strength resulting from changes in option selection, increasing the choice-set size also affected how long females took to make the decision, and whether they failed to make one in the allotted time (deferred). Females took longer to choose and were less likely to make a choice as the number of available options increased, particularly in the largest choice set (eight choices). While decision latency largely depended on the number of options, we also found that females in quiet conditions took longer to decide in two-choice trials, whereas under noise, they took longer in eight-choice trials. This suggests that background noise modified the relationship between task complexity and decision time, reducing latency differences across option numbers under quiet conditions but amplifying them under noise. While decision latency often reflects a speed-accuracy tradeoff,^42^ our results indicate that increased decision times did not improve choice accuracy with more males present, at least at the population level. Longer listening times do improve available information about male call traits, but do not “rescue” decision-making performance in noise.^43^ Although modest, the longer decision times observed here may be biologically relevant. Lekking presents an especially risky environment because mate searching, signaling, and copulation can each be exploited by predators and parasitoids.^44^^,^^45^ Even small delays in decision-making can have fitness consequences by increasing exposure to predators or parasites or an increase in energetic expenditure while females attend the chorus. Importantly, we found delays here even in this relatively simple experimental scenario, in which we simplified the acoustic and spatial complexity relative to natural breeding aggregations. In natural leks, where females may encounter hundreds or thousands of signaling males across larger spatial scales, even modest increases in decision time could accumulate. The extent to which these temporal effects scale to influence female fitness in natural choruses remains an open question. Future work could empirically test these ideas by tracking females across natural choruses to determine whether latency and mating probability vary with chorus size and composition.

If choice overload interferes with the expression of female mating preferences, it may also shape male signaling strategies in this species. That female preference expression declines as the number of calling males increases suggests that group size, rather than only the properties of individual signals, may be a critical factor shaping male mating success. Because *H. chrysoscelis* and its relatives breed in dense congregations, the spatial relationships among signalers may matter on a local scale. Much previous research has addressed why choruses form and what the dynamics within the chorus are; hypotheses, including the “hotshot” and “hotspot” models, have sought to explain the local arrangement of males in the chorus.^46^ Recent evidence from the sister taxon *H*. *versicolor* showed that females prefer to approach small clusters of males over single calling individuals.^47^ This, combined with the data presented in this paper, suggests that individual females may prefer to choose from bigger groups of males, even at the cost of reduced ability to discriminate among individuals. Whether males benefit or suffer from these conditions may depend on male quality. When more males aggregate to attract females, the difference in how many mates each male gets often becomes smaller,^48^ suggesting that attractive males may gain a competitive advantage by joining smaller groups, where their preferred call traits can be more easily detected and evaluated by females.^49^^,^^50^^,^^51^ In contrast, males with less attractive calls may benefit from chorusing in larger aggregations, where the sheer abundance of males may reduce the strength of female preferences, leveling the playing field and increasing their probability of success.^7^^,^^16^^,^^50^^,^^51^ Nonetheless, females may still benefit from visiting larger groups, as they may provide greater diversity, reduce travel time between leks, and facilitate more direct comparisons between potential mates.^6^^,^^47^ Our findings highlight how variation in male aggregation behavior may represent an adaptive response to context-dependent trade-offs between signal detectability and female decision-making capacity in complex acoustic environments. A mathematical model-based approach may allow us to explore the fitness trade-offs males face when advertising in larger vs. smaller groups, depending on their relative attractiveness and female preferences for larger groups.

We found that females showed similar performance in the presence versus absence of noise across all choice set sizes (two, four, and eight options). This result was somewhat unexpected. Noise had little effect on female preference in our two-choice set. This result was consistent with previous studies showing limited noise effects on call duration preference when receivers chose between a call duration close to the population mean and an alternative two standard deviations lower.^24^^,^^30^^,^^35^^,^^36^ However, we expected that noise would interact with choice set size and reduce performance in our treatments with four and eight alternatives. Instead, with eight options, females exhibited slightly higher accuracy. One explanation could involve a speed-accuracy trade-off: females took longer to decide under noise in the eight-choice tests, which may have facilitated more deliberate processing and increased precision. Another, non-mutually exclusive possibility is that noise facilitated signal processing. In frogs, peripheral and central auditory function is often enhanced in the presence of low-to-moderate levels of noise.^52^^,^^53^^,^^54^^,^^55^ Importantly, behavioral experiments also show that, under certain circumstances, background noise may improve call detection by male frogs^53^^,^^55^ and call discrimination by female frogs.^56^ Our results add to the few studies showing that, under certain conditions, noise may interfere little with or even facilitate signal processing and discrimination in frogs. How neurophysiological processes associated with signal detection in noise correspond to receivers’ perceptual abilities to discriminate communication signals, and whether they can potentially counteract the cognitive load imposed by multiple options, remains an open question.

Across many decision contexts, including mate choice, animals frequently encounter multiple options simultaneously, and emerging evidence suggests that cognitive constraints that lead to choice overload may play a more substantial role in shaping decision-making than previously considered.^16^ A growing number of studies have demonstrated that choice overload occurs in diverse behavioral contexts, including foraging in bees,^18^ house-hunting in ants,^19^ oviposition sites in mosquitoes,^20^ and mate choice in field crickets and frogs,^7^^,^^8^ indicating that it may represent a fundamental mechanism underlying choice behavior across both choice contexts and taxa.^16^ In acoustically signaling species, research on mate preferences has traditionally emphasized masking in signal detection, recognition, and discrimination (reviewed in the sudy by Lee et al.^15^), yet cognitive limitations may be more strongly influencing how individuals process and evaluate information in some contexts. Recognizing these constraints broadens our understanding of the sensory and cognitive ecology of mating systems and informs how we model selective pressures in natural populations. Furthermore, this perspective may help resolve the long-standing “lek paradox”, or the apparent maintenance of genetic variation despite strong directional sexual selection.^57^ If cognitive constraints prevent receivers from fully expressing preference in more complex environments, then the strength of selection typically observed in controlled laboratory settings using single-stimulus or two-choice tests^3^ likely represents an upper limit of what can occur in nature.

Our results highlight how cognitive constraints can potentially shape the dynamics of sexual selection, independent of energetic masking. When individuals are faced with complex choice environments, limitations in decision-making can influence the expression of mate preferences, which in turn shape selection on sexual signals. Such cognitive constraints likely operate across sensory modalities and taxa, suggesting that choice overload may be a general feature of mating systems where animals aggregate, such as leks, choruses, and swarms. By exploring the cognitive limits involved in mate choice decisions, we can expand our understanding of how information processing interacts with signal design, potentially constraining the elaboration of sexual traits.^1^^,^^5^ Future work integrating cognitive ecology with studies and models of sexual selection will be essential to uncover the extent to which these constraints shape evolutionary trajectories across diverse mating systems.

### Limitations of the study

Our experimental design allowed us to isolate the effects of choice overload from similar predicted effects of energetic masking. However, our controlled laboratory approach has some limitations regarding external validity. Our playback design relied on discrimination between calls with different call durations, but which were otherwise identical. Because of this, we are unable to draw conclusions about whether choice overload may similarly affect mate choice based on other acoustic properties. Additionally, the discrimination task—in which females discriminated between a target and sometimes up to 7 identical distractors—is unusual because, in natural choruses, any two adjacent males are likely to differ in multiple dimensions. We presented calls sequentially without temporal overlap, whereas calls typically overlap in natural choruses as a natural consequence of both the variable call rates within and between males and the large number of males attending the lek. To manipulate background noise, we broadcast chorus-shaped noise from a single overhead speaker, rather than allowing noise to emerge from spatially distributed sound sources (male calls) as in a natural chorus. Additionally, females began each trial exactly equidistant (1 m) from all simulated calling males, which could artificially increase the difficulty of decision-making. On the other hand, we conducted trials in an artificially simple environment, which removed many of the structural impediments (such as vegetation) and risks (exposure to predators) of performing mate choice in nature, which could exacerbate the symptoms of choice overload. These constraints represent necessary trade-offs to achieve the experimental isolation of choice overload, which is the primary strength of our design.

## Resource availability

### Lead contact

Further information and requests for resources should be directed to and will be fulfilled by the lead contact, Claire Hemingway (chemingw@utk.edu).

### Materials availability

This study did not generate unique reagents.

### Data and code availability

• All datasets for behavioral experiments and setup are publicly available on Mendeley Data:^58^ https://data.mendeley.com/datasets/mgpbwfztws/1.
• R scripts and MATLAB scripts for setup and analysis are publicly available on Mendeley Data^58^ (https://data.mendeley.com/datasets/mgpbwfztws/1).
• Any additional information required to reanalyze the data reported in this paper is available upon request from the lead contact.

## Acknowledgments

We are grateful to all members of the 2025 Tanner lab, especially Trina Chou, Victoria Nalls, Taylor McClain, and Sasha Ewing, for collecting frogs, making recordings of natural frog choruses, performing animal care, and assisting with behavioral experiments. Joe Hinton and family generously provided access to their property in South Knoxville. The Tennessee Wildlife Resources Agency, especially Rusty Boles, were helpful with permitting and establishment of lab protocols. We thank the staff of the North Cumberland Wildlife Management Area for access to field sites. Funding was provided by the 10.13039/100007135University of Tennessee to C.T.H., A.V., and J.C.T.

## Author contributions

All authors conceived of the study and designed experiments. A.V.R. and J.C.T. generated synthetic frog calls. A.V. and J.C.T. generated the chorus-shaped noise. A.V.R. and J.C.T. performed the experiment. A.V.R. analyzed the data. A.V.R. and J.C.T. generated the figures. C.T.H. and A.V.R. wrote the original draft with input from A.V. and J.C.T. All authors edited the manuscript and approved the final version.

## Declaration of interests

The authors declare no competing interests.

## STAR★Methods

### Key resources table

**Table:** 

REAGENT or RESOURCE	SOURCE	IDENTIFIER
**Deposited data**		

Data, setup, and analysis code	This paper	https://doi.org/10.17632/mgpbwfztws.1

**Software and algorithms**		

MATLAB (version 2025a)	Mathworks	https://www.mathworks.com/products/matlab.html
RStudio (version 2025.4.5.0)	RStudio	https://www.r-project.org/

### Experimental model and study participant detail

#### Ethical note

All procedures described here met with the ABS/ASAB Guidelines for the Treatment of Animals in Behavioral Research and Teaching (ASAB Ethical Committee/ABS Animal Care Committee, 2023) and were approved by the University of Tennessee Institutional Animal Care and Use Committee (IACUC) under Protocol number 2957–0123. Animals were collected under Scientific Collection Permit no. 6756 from the Tennessee Wildlife Resources Agency.

#### Subjects

We collected 60 gravid females of *H. chrysoscelis* in amplexus with males from breeding choruses at Butterfly Lake (35.9197985, −83.879307; *n* = 13) or North Cumberland Wildlife Management Area (36.3597583, −84.2237885; *n* = 47) in East Tennessee from late spring to early summer between 21:30 and 00:00. Females caught in amplexus are just as discriminating as female frogs caught prior to mate choice.^59^ Pairs were transported to the laboratory; provisioned with clean, aged tap water; and their containers were stored on ice to delay egg deposition. Treefrogs typically drop their eggs within several hours of collection if stored at room temperature, after which they are not phonotactic (i.e., they do not move toward sounds they recognize as conspecific calls). Immediately before testing, pairs were removed from ice, supplied with room temperature water, and placed in an incubator at 20°C for at least 30 min. Between trials, females were returned to their containers and housed with their mates for a time out period of at least 3 min and up to 12 min. Each female finished their battery of tests within about 3 h of being removed from ice. Upon finishing the series of 12 tests, pairs were housed together at room temperature until they were released at their capture locations within 3 days of collection.

### Method details

#### Experimental design and playback

**Figure 2:**
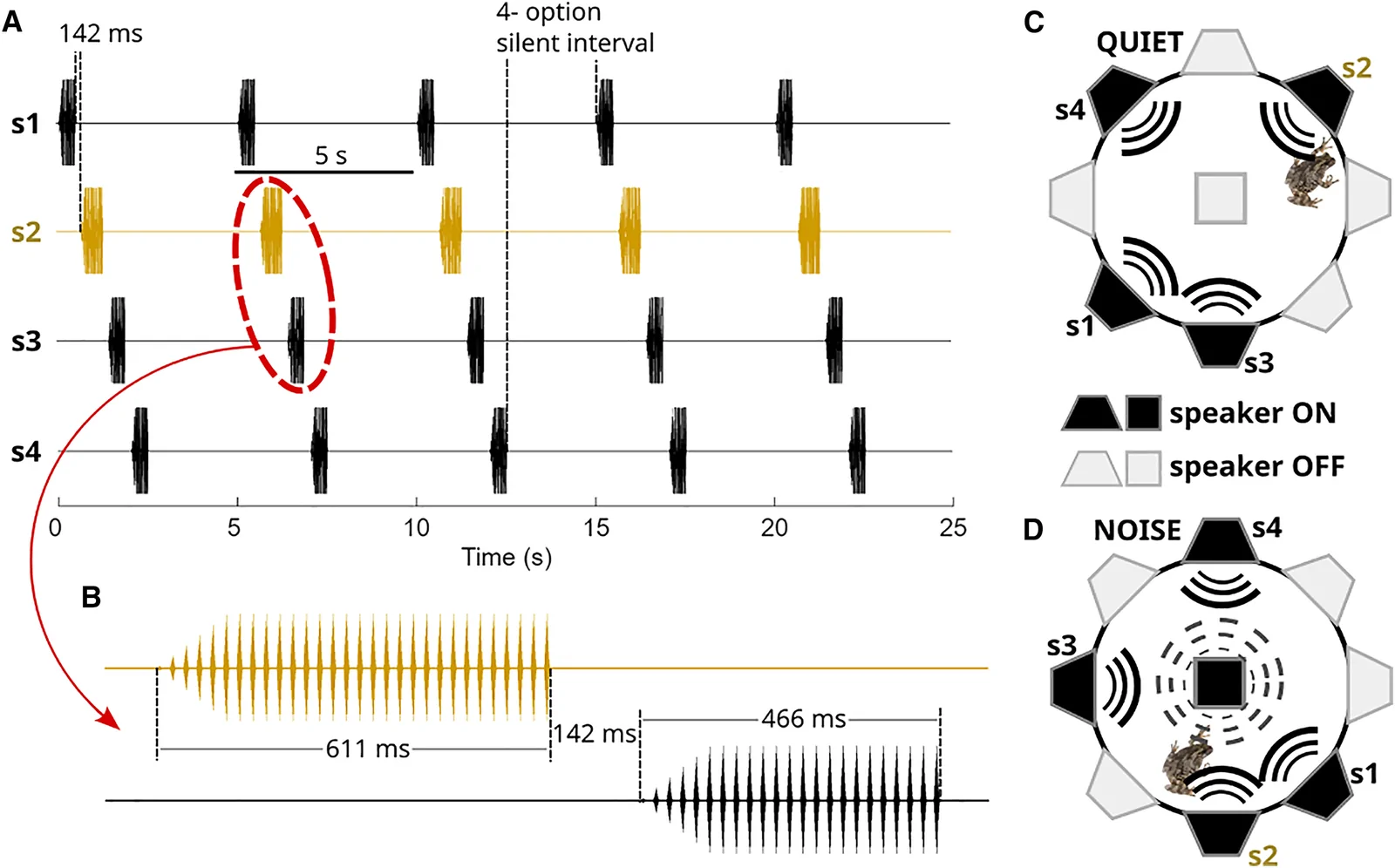
Illustration of experimental design for a 4-option example phonotaxis test (A) Waveforms showing four non-overlapping acoustic stimuli (s1-s4) shown in their temporal arrangement (s2: target in gold; s1, s3, and s4: distractors in black). (B) Detailed view of two consecutive calls (one target and one distractor), showing pulse structure and number, call duration, and inter-call intervals. (C) Overhead view of the 1-m circular arena in a 4-option trial in the quiet condition. Active speakers in black broadcast the male calls (s1 to s4); inactive speakers in light gray. The central overhead speaker used for the noise treatment is inactive. (D) Overhead view of the arena in the noise condition, with the central (overhead) noise-broadcasting speaker active. Note the different randomized spatial arrangement of the speakers (cf. C and D); each subject was assigned to two different randomized spatiotemporal locations of the target and distractors.

We presented receivers with a choice between one longer-duration "target" call and one, three, or seven shorter-duration "distractor" calls, and we examined how the number of call options and background noise influenced female gray treefrog phonotaxis. Calls were presented sequentially, such that only one call was broadcast at any given moment, eliminating temporal overlap (Figures 2A and 2B). We measured three behavioral responses: 1) choice accuracy, the probability of choosing the target call; 2) latency to choose, the time elapsed before making a choice; and 3) choice deferral, the probability of not responding during the allotted time. Each female was tested across all combinations of option number (two, four, or eight) and background noise condition (quiet or noise), yielding six unique treatment combinations. Between subjects, we randomized the location of the target call speaker. In the eight-option tests, all remaining speakers broadcast distractor calls. In the two- and four-stimulus tests, we randomized which speakers were used and which remained silent in order to control for potential effects of spatial relationships between target and distractor locations. Additionally, we randomized the temporal order of call onset so that the target call could occur in any order within the presentation sequence, from first to last. Because we wished to control the time elapsed between any two subsequent call presentations to account for any possibility of forward masking,^60^ we did not allow empty “timeslots”; instead, in the 2- and 4- choice tests, only the first two and four timeslots were used, respectively. This means that, in eight-choice tests, calls were presented sequentially and repeated in order with equal-length pauses between consecutive calls (e.g., D1 D2 D3 T4 D5 D6 D7 D8), whereas in two- and four-choice tests only the first two or four temporal positions were filled, and the remaining positions were left silent before repeating the sequence (Figures 2A and S1). Each female was tested with two randomized combinations of spatial and temporal relationships between the target and distractor(s), resulting in 12 trials. Fifty-two females completed all 12 trials while eight females completed a subset (n_trials_ = 675; n_females_ = 60; Table S9).

Playback experiments were conducted in a semi-anechoic chamber (3.86 × 3.40 × 2.59 m; IAC Acoustics, North Aurora, IL) whose walls were constructed from “Planarchoic” acoustic paneling. Females moved freely under infrared light on interlocking foam mats that covered the floor. We monitored female behavior from outside the chamber using an infrared camera (ELP 1080p, wide-angle fisheye) illuminated by an IR light source (Tenderlux AI4, 4 W). Inside the chamber, we constructed an acoustically transparent testing arena from hardware cloth covered with black cloth. The arena was shaped as a 1-m-radius circle, with eight equidistant speakers (Orb Audio Mod 1) placed on the floor, evenly spaced around the perimeter. This arrangement resulted in a 45-degree angular separation between the speakers, sufficient for gray treefrog females to discriminate between and localize signals.^7^^,^^61^^,^^62^ Synthetic advertisement calls were broadcast at 85 dB SPL (L_max_, C-weighted) at 1 m, calibrated with a Larson Davis SoundAdvisor 831C sound level meter and a PCB Piezotronics microphone (model 377B02) placed in the center of the arena. Background noise was broadcast from a ceiling-mounted speaker 2 m above the center of the arena and calibrated to 70 dB SPL (L_eq_, C-weighted) over a 1-min period measured at the frog’s starting position on the floor of the arena (Figures 2C and 2D). This approach has been used previously to introduce masking noise in phonotaxis experiments.^63^^,^^64^^,^^65^^,^^66^

At the start of each trial, a female was placed in an acoustically transparent release cage (10 cm diameter and 3 cm height) constructed from plastic mesh at the arena center and allowed 1 min to acclimate in silence, followed by 30 s of stimulus playback. The trial began when the cage was lifted, allowing free movement within the arena, and ended when the female entered a 10-cm radius response zone in front of a speaker or after 10 min had elapsed without a choice being made.^7^^,^^67^^,^^68^^,^^69^ Between trials, females were returned to containers with males and placed into an incubator held at 20 °C. The rest period between trials lasted between 3 and 12 min, as females were tested in alternating pairs. Females that did not respond in two consecutive trials were given a “reference test” to confirm receptivity, similar to other published studies.^63^^,^^67^ In reference tests, a single “standard” call was broadcast (detailed below). Females that did not respond to the standard call within 5 min (*n* = 8) were not tested further, but we retained all data up to their last phonotactic response.

#### Stimuli design

We generated synthetic male advertisement calls *de novo* from modified cosine waves using Synsing Graphical User Interface^64^^,^^68^ in MATLAB version 2025a (the Mathworks, Natick, MA). Receivers were presented with two types of stimuli: a "target" (30 pulses per call) and some number (either 1, 3, or 7) of ‘distractors’ (23 pulses per call) (Figure S1). Previous studies consistently show that gray treefrog receivers prefer calls with more pulses^30^^,^^31^; thus, we designated the longer call as the ‘target’ and shorter ones as "distractors". Our results in the two-choice tests and the absence of background noise also confirmed a strong population-level preference for the longer call (Figure 1A). Both target and distractor calls had two spectral peaks at 1300 Hz (−4 dB) and 2600 Hz (0 dB). Individual pulses were 10.4 ms in duration, separated by 10.4 ms inter-pulse intervals, resulting in a pulse rate of 48.281 pulses/s. Target calls consisted of 30 pulses (611.004 ms), each followed by a 4.4 s inter-call interval to achieve an approximately population-mean call rate (∼12 calls/min). This target call closely matches the population mean (30 pulses vs. a mean of 31.6 pulses per call ±7.7 s d at 20°C, Tanner, unpublished data). Distractor calls consisted of 23 pulses (466.02 ms), with a 4.545 s inter-call interval (∼12 calls/min). This is approximately one standard deviation below the population mean. Pulses were shaped with rise times of 4.855 ms (41%–50% amplitude) and fall times of 5.5 ms (25.5%–50% amplitude). Calls were shaped with a 110 ms rise to 50% amplitude and no defined fall shaping. We concatenated the calls with appropriate inter-call intervals to generate 11 min of calls. Target and distractor stimuli were identical except for call duration (Figures 2A and 2B).

Because female gray treefrogs strongly prefer faster call rates,^7^^,^^23^^,^^31^ we maintained identical call rates for targets and distractors, allowing inter-call intervals to vary to accommodate differences in call duration between target and distractor. We could thus interleave up to eight non-overlapping stimuli (one target and seven distractors) while leaving a 142.25 ms silent interval between calls, sufficient to prevent forward masking in gray treefrogs.^60^

**Figure 3:**
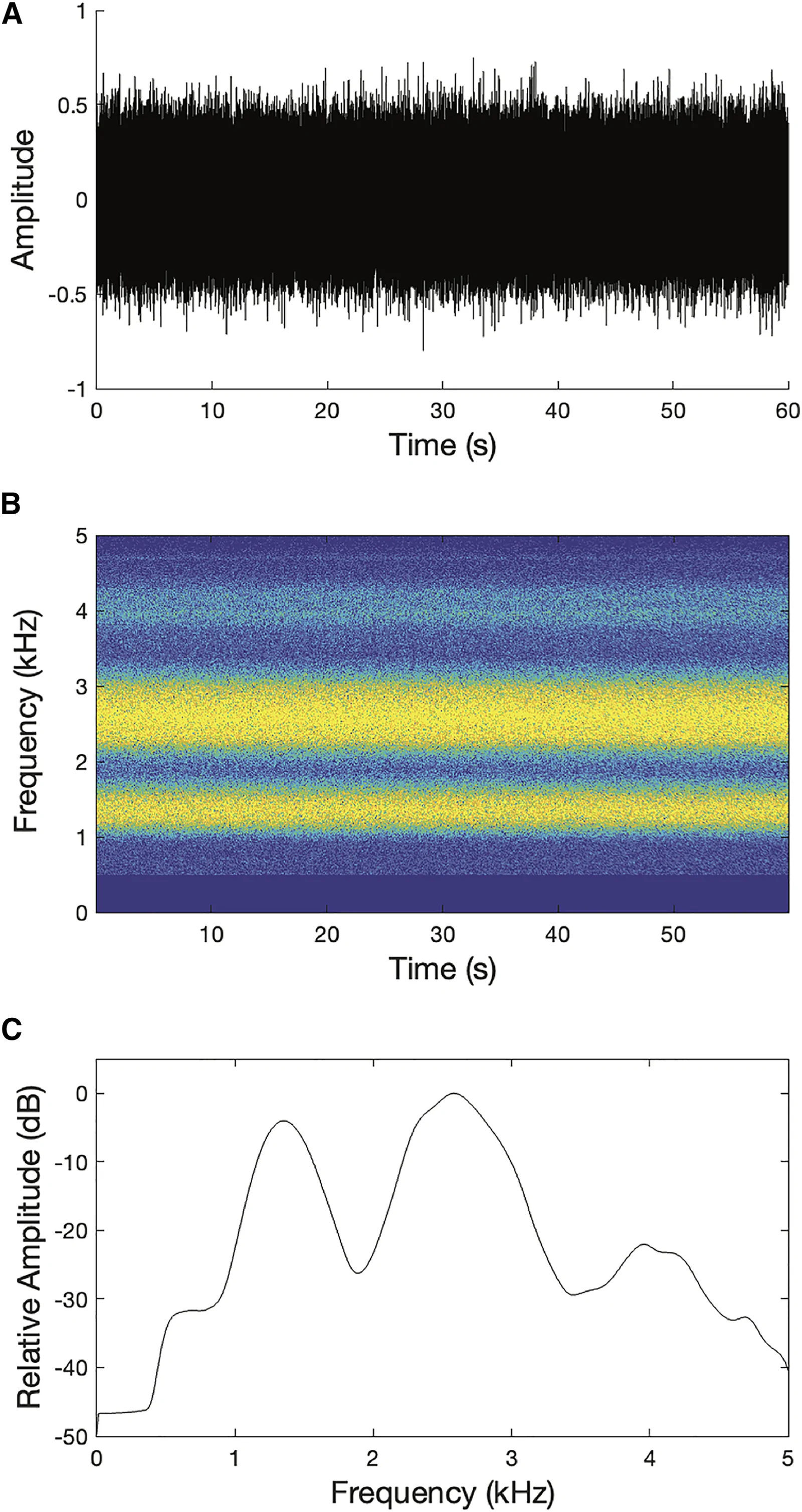
Background noise played back in the noise condition was designed to have the long-term spectral characteristics of a natural gray treefrog chorus (A) The waveform (top left) shows the amplitude over time for a representative 1-min sample of noise. (B) Spectrogram shows frequency (kiloHertz [kHz]) and intensity (color) over time (seconds). (C) The power spectrum shows the distribution of energy, measured as relative amplitude (decibels [dB]), across frequency (kHz). Gray treefrog calls typically have energy “peaks” in two main frequency bands: the dominant at approximately 2.5 kHz and the fundamental at approximately 1.25 kHz, which are visible as the yellow bands in (B) and the two tallest peaks in (C).

To generate the noise with the long-term spectral characteristics of a treefrog chorus,^63^ we used recordings of natural choruses at our field sites. We recorded 5-min samples of nine natural choruses near peak calling activity (2230-0030h) using a Sennheiser ME-62 omnidirectional microphone and a Tascam DR-40X recorder at a sampling rate of 44.1 kHz and a bit depth of 16. In MATLAB version 2023b, we calculated the spectral envelope for a 90 s sample of each chorus recording and used it to create a filter through which we passed white noise. We then corrected the chorus-shaped noise in the playback environment by filtering with the inverse of the spectral envelope of a recording of white noise played through the speaker used to broadcast the noise. The resulting chorus-shaped noises captured the acoustic energy of natural treefrog choruses (Figure 3).

#### “Standard” stimulus used in reference tests

If a receiver did not respond in two consecutive tests, we conducted a reference test in which we played back a single, standardized “reference” stimulus expected to reliably elicit phonotactic responses. This procedure, carried out in the absence of background noise, allowed us to ensure females were still sexually receptive, which is crucial to distinguishing between cases where receivers did not respond due to choice overload or the interference of noise (a valid outcome in our experiment) and cases where receivers did not respond because they were no longer receptive (in which case we discontinued testing).

The reference stimulus was generated using Synsing Graphical User Interface^70^^,^^71^ in MATLAB, similar to the other stimuli described in the main text. The acoustic parameters were designed to have approximately population-mean characteristics for all call traits, including a call rate of 12.8 calls per minute, 32 pulses per call, and a pulse rate of 48.3 pulses per second with a 50% pulse duty cycle.

### Quantification and statistical analysis

Analyses were conducted in R (version 2025.4.5.0). Our three main responses were choice accuracy (choice for the target option), latency to choose, and the probability of deferring choice - all behaviors predicted to change under choice overload.^17^ We fitted three GLMMs (Formulae S1) using *glmmTMB*^72^ to analyze target choice (binomial), latency (gamma, log link), and deferral probability (binomial). For all models, we included the number of options (two, four, eight), noise condition (quiet/noise), and their interaction as fixed effects. Because our experiment was organized into stimulus sets, each containing six treatments, with females completing two sets per treatment (12 trials), we included random intercepts for the stimulus set and for female identity nested within the stimulus set (Formula S1). For all response variables, we tested *a priori* for effects of trial number (Table S6, see below). To assess choice accuracy, we tested *a priori* for effects of temporal order (Table S7) and spatial position (Tables S8 and S9, see below). Pairwise contrasts were computed with emmeans^73^ and a false discovery rate (FDR) correction to control for the increased risk of false positives (see Formulae S1 for all final models).

#### Model covariates

Female behavior may change across the sequence of trials. We found that later trials were associated with decreased latency and decreased accuracy, and showed a non-significant trend toward reduced deferral (Table S5). This decline suggests females became more responsive and less selective as they approached oviposition. Accordingly, trial number was included as a covariate in all models (Formulae S1).

In some frog species, the first call in a sequence can disproportionately attract phonotactic responses.^4^ We therefore tested whether the temporal position of the target call predicted choice accuracy separately for each choice number. Females were more likely to choose the target when it occurred first in the temporal sequence during the two- and four-option tests, whereas no such pattern was apparent in the eight-option tests, where calls cycled continuously (Table S6). Thus, temporal position was included as a covariate only in the accuracy model (Formula 1C).

We also randomized speaker use in two- and four-option tests. Because females might either prefer clusters of males over spatially isolated calls^1^ or because it is conceivable that sounds clustered in space might be more difficult to discriminate than sounds separated in space, we tested for effects of speaker position. Specifically, we asked whether receiver responses were different if the target speaker’s nearest 1 or 2 neighbors were broadcasting distractors, compared to when the target speaker’s nearest neighboring speakers were not in use. The arena contained eight fixed speaker positions, with equidistant speakers spaced 45° apart around the perimeter and facing the center. We tested whether the spatial clustering of calls (Table S7) or the angular separation between target and distractors affected behavior and found no significant effects (Table S8), so these terms were not included in the final model.

#### Accounting for chance expectations

While we can compare accuracy directly across choice-set sizes, how far above chance levels receivers perform is not directly comparable across tests with different numbers of options because the probability of chance selection decreases as the number of alternatives increases (e.g., 0.5 for two options, 0.25 for four options, and 0.125 for eight options). To account for this, we first obtained the GLMM estimates and their 95% confidence intervals on the logit scale and subtracted the expected probability by chance. This produced an adjusted distribution of target choices relative to chance. Using these adjusted distributions, we used a Monte Carlo simulation to generate 10,000 random samples per treatment. Pairwise differences were computed by subtracting the simulated draws between treatments (e.g., two-quiet minus two-noise, or two-quiet minus four-quiet). For each contrast, we calculated the proportion of simulated differences greater than zero. These proportions provided a probabilistic measure of whether one treatment’s response exceeded the other’s.

## Supplemental information

Document S1. Figures S1 and Tables S1–S9 and Formulae S1

## References

[1] Bateson M., Healy S.D. (2005). Comparative evaluation and its implications for mate choice. Trends Ecol. Evol..

[2] Ryan M.J., Akre K.L., Kirkpatrick M., Dukas R., Ratcliffe J.M. (2009). Cognitive Ecology II.

[3] Dougherty L.R. (2020). Designing mate choice experiments. Biol. Rev..

[4] Gerhardt H., Huber F. (2022).

[5] Ryan M.J., Cummings M.E. (2013). Perceptual biases and mate choice. Annu. Rev. Ecol. Evol. Syst..

[6] Hutchinson J.M.C. (2005). Is more choice always desirable? Evidence and arguments from leks, food selection, and environmental enrichment. Biol. Rev..

[7] Tanner J.C., Shein-Idelson M., Hoke K.L., Bee M.A. (2025). Complex choice environments shelter unattractive signallers from sexual selection. Proc. R. Soc. A B..

[8] Tanner J.C., Simmons L.W. (2022). Spoiled for choice: number of signalers constrains mate choice based on acoustic signals. Behav. Ecol..

[9] Culling J.F., Stone M.A., Middlebrooks J.C., Simon J.Z., Popper A.N., Fay R.R. (2017). The Auditory System at the Cocktail Party Springer Handbook of Auditory Research.

[10] Brumm H., Slabbekoorn H. (2005). Acoustic communication in noise. Adv. Study Behav..

[11] Branstetter B.K., Sills J.M. (2022). Mechanisms of auditory masking in marine mammals. Anim. Cogn..

[12] Lohr B., Wright T.F., Dooling R.J. (2003). Detection and discrimination of natural calls in masking noise by birds: estimating the active space of a signal. Anim. Behav..

[13] Bee M.A., Micheyl C. (2008). The cocktail party problem: What is it? How can it be solved? And why should animal behaviorists study it?. J. Comp. Psychol..

[14] Römer H., Brumm H. (2013). Animal Communication and Noise Animal Signals and Communication.

[15] Lee N., Vélez A., Bee M. (2023). Behind the mask(ing): how frogs cope with noise. J. Comp. Physiol..

[16] Tanner J.C., Hemingway C.T. (2025). Choice overload and its consequences for animal decision-making. Trends Cogn. Sci..

[17] Chernev A., Böckenholt U., Goodman J. (2015). Choice overload: A conceptual review and meta-analysis. J. Consum. Psychol..

[18] Austin M.W., Horack P., Dunlap A.S. (2019). Choice in a floral marketplace: the role of complexity in bumble bee decision-making. Behav. Ecol..

[19] Sasaki T., Pratt S.C. (2012). Groups have a larger cognitive capacity than individuals. Curr. Biol..

[20] Sharma M., Isvaran K. (2023). Spoilt for choice: Do female mosquitoes experience choice overload when deciding where to lay eggs?. Behav. Processes.

[21] Gerhardt H.C. (1975). Sound pressure levels and radiation patterns o the vocalizations of some North American frogs and toads. J. Comp. Physiol..

[22] Halfwerk W., Lea A.M., Guerra M.A., Page R.A., Ryan M.J. (2016). Vocal responses to noise reveal the presence of the Lombard effect in a frog. Behav. Ecol..

[23] Tanner J.C., Bee M.A. (2019). Within-individual variation in sexual displays: signal or noise?. Behav. Ecol..

[24] Vélez A., Schwartz J.J., Bee M.A., Brumm H. (2013). Animal communication and noise.

[25] Bishop P.J., Jennions M.D., Passmore N.I. (1995). Chorus size and call intensity: Female choice in the painted reed frog, *Hyperolius marmoratus*. Beyond Behav..

[26] Gerhardt H.C. (1987). Evolutionary and neurobiological implications of selective phonotaxis in the green treefrog, *Hyla cinerea*. Anim. Behav..

[27] Schwartz J., Buchanan B., Gerhardt H. (2002). Acoustic interactions among male gray treefrogs, *Hyla versicolor*, in a chorus setting. Behav. Ecol. Sociobiol..

[28] Taylor R.C., Wilhite K.O., Ludovici R.J., Mitchell K.M., Halfwerk W., Page R.A., Ryan M.J., Hunter K.L. (2020). Complex sensory environments alter mate choice outcomes. J. Exp. Biol..

[29] Jannasch L.L., Grüb J., Nieder A. (2025). Shared cognitive biases influence numerical judgments in macaques and crows. Proc. Natl. Acad. Sci. USA.

[30] Bee M.A. (2008). Parallel female preferences for call duration in a diploid ancestor of an allotetraploid treefrog. Anim. Behav..

[31] Tanner J.C., Ward J.L., Shaw R.G., Bee M.A. (2017). Multivariate phenotypic selection on a complex sexual signal. Evolution.

[32] McGregor P., Horn A., Leonard M., Thomsen F., Brumm H. (2013). Animal Communication and Noise.

[33] Nityananda V., Bee M.A. (2011). Finding your mate at a cocktail party: Frequency separation promotes auditory stream segregation of concurrent voices in multi-species frog choruses. PLoS One.

[34] Vélez A., Linehan-Skillings B.J., Gu Y., Sun Y., Bee M.A. (2013). Pulse-number discrimination by Cope’s gray treefrog (*Hyla chrysoscelis*) in modulated and unmodulated noise. J. Acoust. Soc. Am..

[35] Ward J.L., Love E.K., Vélez A., Buerkle N.P., O’Bryan L.R., Bee M.A. (2013). Multitasking males and multiplicative females: dynamic signalling and receiver preferences in Cope’s grey treefrog. Anim. Behav..

[36] Schwartz J.J., Buchanan B.W., Gerhardt H.C. (2001). Female mate choice in the gray treefrog (*Hyla versicolor*) in three experimental environments. Behav. Ecol. Sociobiol..

[37] Sullivan B.K., Hinshaw S.H. (1992). Female choice and selection on male calling behaviour in the grey treefrog *Hyla versicolor*. Anim. Behav..

[38] Hebets E.A., Papaj D.R. (2005). Complex signal function: developing a framework of testable hypotheses. Behav. Ecol. Sociobiol..

[39] Tumulty J.P., Bee M.A. (2021). Ecological and social drivers of neighbor recognition and the dear enemy effect in a poison frog. Behav. Ecol..

[40] Schul J. (1998). Song recognition by temporal cues in a group of closely related bushcricket species (genus *Tettigonia*). J. Comp. Physiol. Sensory Neural Behav. Physiol..

[41] Schul J., Bush S.L. (2002). Non-parallel coevolution of sender and receiver in the acoustic communication system of treefrogs. Proc. R. Soc. Lond. B..

[42] Chittka L., Skorupski P., Raine N.E. (2009). Speed–accuracy tradeoffs in animal decision making. Trends Ecol. Evol..

[43] Tanner J.C., Bee M.A. (2020). Inconsistent sexual signaling degrades optimal mating decisions in animals. Sci. Adv..

[44] Lima S.L., Dill L.M. (1990). Behavioral decisions made under the risk of predation: a review and prospectus. Can. J. Zool..

[45] Zuk M., Kolluru G.R. (1998). Exploitation of sexual signals by predators and parasitoids. Q. Rev. Biol..

[46] Beehler B.M., Foster M.S. (1988). Hotshots, hotspots, and female preference in the organization of lek mating systems. Am. Nat..

[47] Stratman K.D., Oldehoeft E.A., Höbel G. (2021). Woe is the loner: Female treefrogs prefer clusters of displaying males over single “hotshot” males. Evolution.

[48] Kokko H., Sutherland W.J., Lindström J., Reynolds J.D., Mackenzie A. (1998). Individual mating success, lek stability, and the neglected limitations of statistical power. Anim. Behav..

[49] Cestari C., Loiselle B.A., Pizo M.A. (2016). Trade-Offs in Male Display Activity with Lek Size. PLoS One.

[50] Durães R., Loiselle B.A., Parker P.G., Blake J.G. (2009). Female mate choice across spatial scales: influence of lek and male attributes on mating success of blue-crowned manakins. Proc. R. Soc. A B..

[51] Johnstone R.A., Earn D.J.D. (1999). Imperfect female choice and male mating skew on leks of different sizes. Behav. Ecol. Sociobiol..

[52] Nadrowski B., Martin P., Jülicher F. (2004). Active hair-bundle motility harnesses noise to operate near an optimum of mechanosensitivity. Proc. Natl. Acad. Sci. USA.

[53] Narins P., Benedix J., Moss F. (1997).

[54] Penna M., Gormaz J.P., Narins P.M. (2009). When signal meets noise: immunity of the frog ear to interference. Naturwissenschaften.

[55] Ratnam R., Feng A.S. (1998). Detection of Auditory Signals by Frog Inferior Collicular Neurons in the Presence of Spatially Separated Noise. J. Neurophysiol..

[56] Schwartz J.J., Gerhardt H.C. (1998). The neuroethology of frequency preferences in the spring peeper. Anim. Behav..

[57] Kotiaho J.S., Simmons L.W., Tomkins J.L. (2001). Towards a resolution of the lek paradox. Nature.

[58] Hemingway C.T., Rodrigues A.V., Vélez A., Tanner J.C. Data and code from: Choice overload constrains mate choice independent of energetic masking: Cognitive limits on mate choice. Version V1 (Mendeley Data).

[59] Murphy C.G., Gerhardt C.H. (1996). Evaluating the design of mate-choice experiments: the effect of amplexus on mate choice by female barking treefrogs, *Hyla gratiosa*. Anim. Behav..

[60] Hillery C.M., Fay R.R. (1982). Forward masking and suppression in the midbrain of the southern grey treefrog (*Hyla chrysoscelis*). J. Comp. Physiol..

[61] Caldwell M.S., Bee M.A. (2014). Spatial hearing in Cope’s gray treefrog: I. Open and closed loop experiments on sound localization in the presence and absence of noise. J. Comp. Physiol..

[62] Caldwell M.S., Lee N., Schrode K.M., Johns A.R., Christensen-Dalsgaard J., Bee M.A. (2014). Spatial hearing in Cope’s gray treefrog: II. Frequency-dependent directionality in the amplitude and phase of tympanum vibrations. J. Comp. Physiol..

[63] Bee M.A., Christensen-Dalsgaard J. (2016). Sound source localization and segregation with internally coupled ears: the treefrog model. Biol. Cybern..

[64] Vélez A., Bee M.A. (2010). Signal recognition by frogs in the presence of temporally fluctuating chorus-shaped noise. Behav. Ecol. Sociobiol..

[65] Zuk M., Tanner J.C., Schmidtman E., Bee M.A., Balenger S. (2017). Calls of recently introduced coquí frogs do not interfere with cricket phonotaxis in Hawaii. J. Insect Behav..

[66] Reichert M.S., Ronacher B. (2015). Noise affects the shape of female preference functions for acoustic signals: Female preferences in nosie. Evolution.

[67] Larter L.C., Ryan M.J. (2024). Female preferences for more elaborate signals are an emergent outcome of male chorusing interactions in túngara frogs. Am. Nat..

[68] Bastien B., Farley G., Ge F., Malin J.S., Simon-Plumb C.L., Pulley D.M., Yang C., Baugh A.T. (2018). The waiting and mating game: Condition dependent mate sampling in female gray treefrogs (*Hyla versicolor*). Front. Ecol. Evol..

[69] Neelon D.P., Rodríguez R.L., Höbel G. (2019). On the architecture of mate choice decisions: preference functions and choosiness are distinct traits. Proc. R. Soc. A B..

[70] Tanner J.C., Justison J.J., Bee M.A. (2019). Synsing Graphical User Interface (Version 1.0). http://github.com/jessiectanner/SynSing/releases.

[71] Tanner J.C., Justison J., Bee M.A. (2020). SynSing: open-source MATLAB code for generating synthetic signals in studies of animal acoustic communication. Bioacoustics.

[72] Brooks M. (2025). glmmTMB: Generalized Linear Mixed Models Using Template Model Builder.

[73] Lenth R. (2025). Emmeans: Estimated Marginal Means, Aka Least-Squares Means. http://rvlenth.github.io/emmeans.

