# Supplementary material for: Choice overload constrains mate choice independent of energetic masking
Source: iScience. 2026 Aug 12;29(8):117086. doi: 10.1016/j.isci.2026.117086 (PMC13523838; doi:10.1016/j.isci.2026.117086)
Supplement: Supplementary file 1 [file mmc1.pdf]

iScience, Volume *29*

Supplemental information

Choice overload constrains mate choice independent of energetic masking

Claire T. Hemingway, André Vieira Rodrigues, Alejandro Vélez, and Jessie C. Tanner

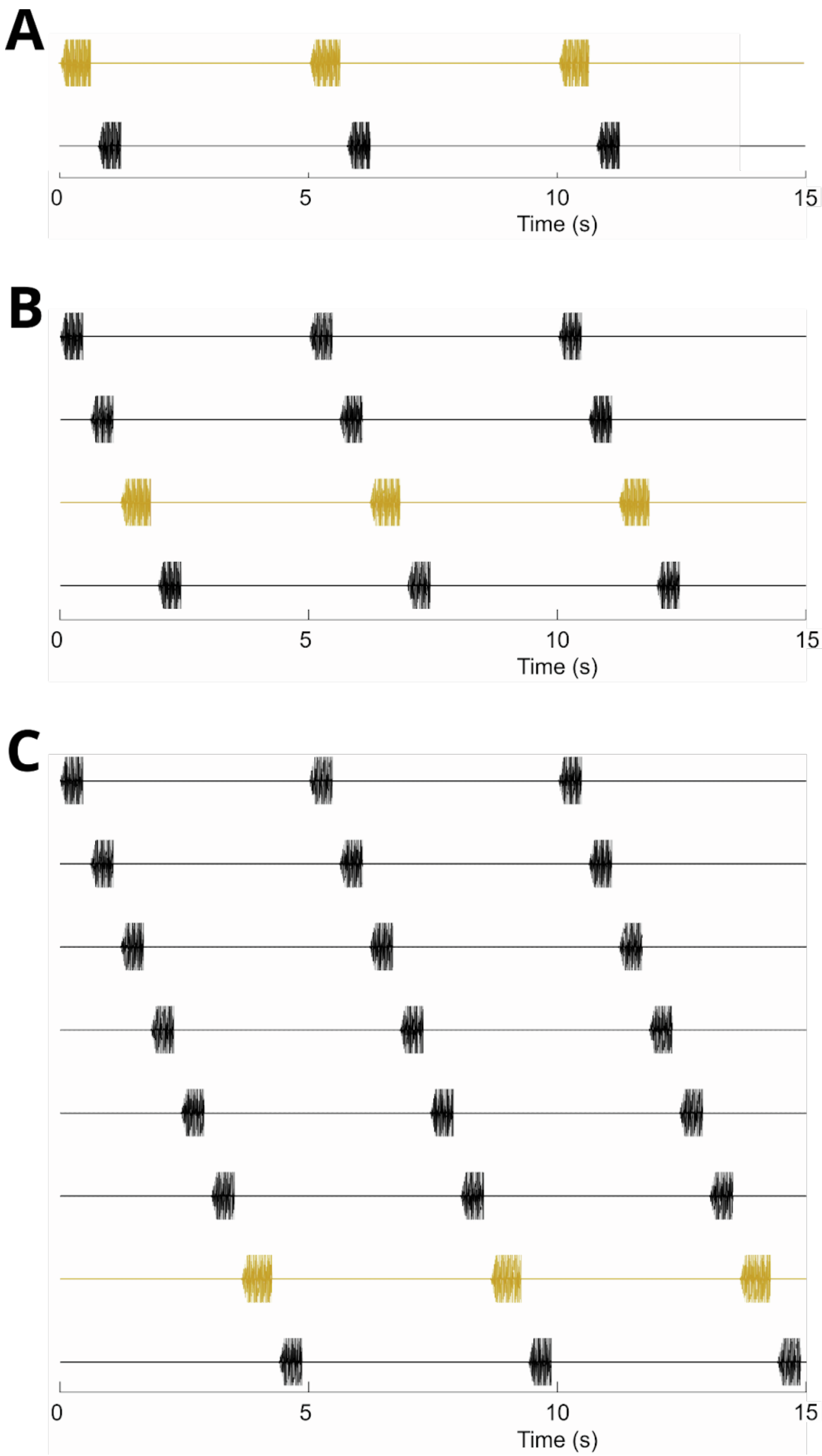

Fig. S1. Example waveforms. Example waveforms (amplitude [y-axis] across time [x-axis]) of concatenated calls showing the temporal arrangements of stimuli used in A) two-, B) four-, and C) eight-choice tests. Target calls rendered in gold are, in these examples, presented in the 1st (A), 3rd (B), and 7th (C) temporal positions, respectively. Distractors are rendered in black.

Table S1: Estimated accuracy (probability of choosing the target) across noise and option-number treatments. Values are derived from model estimates and 95% confidence intervals (CI) from the GLMM with treatment and number of options as fixed effects, and female ID nested within stimulus set as random effects, with sample sizes for each condition. Residual degrees of freedom = 617, Deviance = 726.1

Noise condition	N options	N females	N experiments	Estimate	Lower CI95	Upper CI95
Quiet	2 (0.5)	56	108	0.715	0.598	0.809
	4 (0.25)	56	108	0.454	0.355	0.557
	8 (0.125)	54	97	0.261	0.166	0.386
Noise	2 (0.5)	57	112	0.626	0.509	0.731
	4 (0.25)	57	108	0.386	0.293	0.489
	8 (0.125)	52	94	0.312	0.207	0.440
Covariate			Trial	0.481	0.466	0.496
Covariate			Target order	0.431	0.393	0.470

Table S2: Adjusted-by-chance probability of females choosing the target stimulus across noise and option-number treatments. Values were obtained from post-hoc trans post hoc transformations of GLMM estimates and 95% confidence intervals, with treatment and number of options as fixed effects, and female ID nested within stimulus set as random effects, along with for each condition.

Noise condition	N options	N females	N experiments	Estimate	Lower CI95	Upper CI95
Quiet	2	56	108	0.789	0.692	0.861
	4	56	108	0.725	0.631	0.803
	8	54	97	0.614	0.478	0.734
Noise	2	57	112	0.712	0.608	0.797
	4	57	108	0.667	0.563	0.757
	8	52	94	0.675	0.550	0.779

Table S3: Estimated latencies (in seconds) of females choosing a stimulus across noise and option-number treatments. Values are derived from model estimates (with 95% confidence intervals) from the GLMM with treatment and number of options as fixed effects, and female ID nested within stimulus set as random effects, with sample sizes for each condition. Residual degrees of freedom = 617, Deviance = 7031.0

Noise condition	N options	N females	N experiments	Estimate (s)	Lower CI95 (s)	Upper CI95 (s)
Quiet	2	56	108	106.9	93.5	122
	4	56	108	112.9	98.8	129
	8	54	97	154.3	134.3	177
Noise	2	57	112	89.9	78.9	103
	4	57	108	111.7	97.7	128
	8	52	94	180.7	157	208
Covariate			Trial	0.946	0.928	0.964

Table S4: Estimated probability of choice deferral across noise and option-number treatments. Values are derived from model estimates (with 95% confidence intervals) from the GLMM with treatment and number of options as fixed effects, and female ID nested within stimulus set as random effects, with sample sizes for each condition. Residual degrees of freedom = 666, Deviance = 294.8.

Noise condition	N options	N females	N experiments	Estimate	Lower CI95	Upper CI95
Quiet	2	57	112	0.0121	0.0030	0.0478
	4	56	111	0.0089	0.0019	0.0402
	8	57	112	0.0656	0.0263	0.1543
Noise	2	58	115	0.0084	0.0018	0.0387
	4	58	112	0.0120	0.0029	0.0480
	8	59	113	0.0885	0.0385	0.1907
Covariate			Trial	0.470	0.4359	0.504

Table S5: Effect of trial number. Effect of trial number on choice accuracy, decision latency, and choice deferral.

Behavioral response	Fixed	Estimate	Lower CI95	Upper CI95	Deviance	Df.Residuals
Accuracy	Trial	-0.0627	-0.119	-0.0057	856.5	623
Latency	Trial	-0.0554	-0.0759	-0.0348	7125.9	622
Deferral	Trial	-0.0896	-0.204	0.0248	331.6	671

Table S6: Effect of temporal order. Effect of temporal order of the target call on target-choice accuracy.

Number of calls	Estimate	Lower CI95	Upper CI95	Deviance	Df.Residuals
Two	-3.13	-4.62	-1.64	207.4	216
Four	-0.713	-1.07	-0.356	271.7	212

Eight	-0.0634	-0.215	0.0883	203.1	187

Table S7: Effect of relative spatial position. Effect of relative spatial position (a neighboring distractor speaker) on target-choice accuracy.

Option number	Comparison	Contrast (ratio)	SE	z ratio	p
Two	0 vs. 1 neighbor	1.93	1.3	0.917	0.3313
Four	0 vs. 1 neighbor	0.798	0.350	-0.517	0.8637
	0 vs. 2 neighbor	0.527	0.317	-1.065	0.5358
	1 vs. 2 neighbor	0.661	0.373	-0.734	0.7433

Table S8: Effect of angle separation. Effects of angular separation between target speaker and next distractor speaker on target-choice accuracy.

Option number	Coefficient	Estimate	SE	z value	p
Two	Intercept (Angle = 0º)	1.150	0.777	1.481	0.139
	Angle	0.0058	0.0075	0.775	0.438
Four	Intercept (Angle = 0º)	0.0293	0.515	0.057	0.955
	Angle	-0.0051	0.0077	-0.666	0.505

Table S9: Females used and number of trials completed for each (maximum of 12 trials). Bolded entries indicate the eight females that failed to complete the full trial set.

Female ID	Trials finished	Female ID	Trials finished	Female ID	Trials finished
Hch220-25	12	Hch358-25	12	Hch380-25	12
Hch231-25	12	Hch359-25	12	Hch381-25	12
**Hch232-25**	**4**	Hch360-25	12	Hch382-25	12
Hch280-25	12	Hch361-25	12	Hch383-25	12
Hch282-25	12	Hch362-25	12	Hch388-25	12
Hch283-25	12	Hch363-25	12	Hch389-25	12
Hch284-25	12	Hch364-25	12	Hch390-25	12
Hch285-25	12	Hch365-25	12	Hch392-25	12
**Hch286-25**	**11**	**Hch366-25**	**11**	Hch393-25	12
Hch287-25	12	Hch367-25	12	**Hch398-25**	**3**
Hch291-25	12	Hch368-25	12	Hch399-25	12
Hch292-25	12	Hch369-25	12	Hch404-25	12
Hch295-25	12	Hch370-25	12	Hch405-25	12
Hch296-25	12	Hch371-25	12	Hch406-25	12
**Hch306-25**	**10**	Hch372-25	12	Hch407-25	12
**Hch307-25**	**9**	Hch373-25	12	Hch408-25	12
Hch332-25	12	Hch374-25	12	Hch409-25	12
Hch333-25	12	Hch375-25	12	Hch415-25	12
Hch336-25	12	Hch378-25	12	**Hch416-25**	**2**
Hch337-25	12	Hch379-25	12	**Hch417-25**	**1**

Formulae S1. Formulae of the final models used to examine choice accuracy (A), decision latency (B), and choice deferral (C). Noise presence was a binary factor (0 or 1), while trial.n represented the trial's order (1-12). Target.order represented the target stimulus' temporal position

in the series (in two-choice tests, either 1 or 2; in four-choice tests, either 1, 2, 3, or 4; in eight-choice tests, either 1, 2, 3, 4, 5, 6, 7, or 8). Each female was assigned two distinct and randomly determined combinations of the target's temporal position and the spatial positions of the targets and distractors (stimulus.set) and identified with a unique alphanumeric code (female.id). We therefore fitted female ID nested within stimulus set as a random effect in all models. Models A and C were fitted using the binomial distribution, while model B was fitted using the gamma distribution.

A) **Accuracy (binomial)**

accuracy ~ choice.n * noise.presence + trial.n + target.order + (1 | stimulus.set / female.id)

B) **Latency (gamma)**

latency ~ choice.n * noise.presence + trial.n + (1 | stimulus.set / female.id)

C) **Deferral (binomial)**

deferral ~ choice.n * noise.presence + trial.n + (1 | stimulus.set / female.id)
